# Improving preoperative breast reconstruction consultations: a qualitative study on the impact of personalised audio-recordings

**DOI:** 10.1186/s12905-021-01534-8

**Published:** 2021-11-06

**Authors:** Josipa Petric, Bahara Sadri, Phillipa van Essen, Nicola Ruth Dean

**Affiliations:** 1 Flinders Medical Centre, Flinders Drive, Bedford Park, SA 5042 Australia; 2 College of Medicine and Public Health, Flinders University, Sturt Road, Bedford Park, SA 5042 Australia; 3 Department of Plastic and Reconstructive Surgery, Flinders Medical Centre, Flinders Drive, Bedford Park, SA 5042 Australia

**Keywords:** Breast reconstruction, Referral and consultation, Audio-recordings, Interview, Understanding

## Abstract

**Background:**

To investigate the value of audio-recordings in aiding patient understanding and recall of preoperative breast reconstruction information.

**Methods:**

This was a prospective cohort study. Participants were randomly allocated into either a recording group who were offered the opportunity to record their breast reconstruction explanation of surgery, or a standard information package group who received standard care. The value of having an audio-recording was assessed by semi-structured interviews and analysis of recurring themes.

**Results:**

Between 21/2/19 and 19/3/20, 32 women attending consultations for breast reconstruction consented to participate in the study, 17 were randomly assigned to the recording group and 15 the standard information package group. Twenty-eight of the 32 participants completed qualitative interviews. All participants agreed that audio-recordings were a beneficial resource which allowed them to have a better understanding of the concepts discussed. Commonly reported themes included the ability to listen to the recording multiple times to refresh memory, as well as usefulness in helping to inform other family members. Participants also reported increased levels of trust in their clinician for allowing the audio-recordings. Very few participants raised any medico-legal implications of the recordings, their focus was more on the potential of the audio-recordings to alleviate the overwhelming nature of a pre-operative breast reconstruction consultation.

**Conclusions:**

There was a positive response from participants to the use of audio-recordings in the setting of breast reconstruction consultations. These types of recordings could potentially be used in other complex appointments where detailed information is discussed, with similar success.

**Supplementary Information:**

The online version contains supplementary material available at 10.1186/s12905-021-01534-8.

## Background

It has been previously established that post-mastectomy breast reconstruction is highly effective in improving the wellbeing of women, specifically in terms of improving their psychosocial, physical and sexual well-being [1, 2]. Furthermore, there is evidence that the quality of pre-operative information impacts patients’ perceptions of breast reconstruction and treatment options, as well as their long term satisfaction with results [3–6]. One potential way of increasing patient understanding of the information received in pre-operative consultations is to allow patients an opportunity to make a digital recording of their consultations on their personal devices. In fact, Elwyn and colleagues found that 69% of respondents from the general public desired to record clinical encounters [7]. The largest experience with the use of recorded consultations is in the field of oncology [8] In this field, Benson et al. found that this practice enhanced patient recollection and adherence to management, patients also reported feeling “empowered”. This same group also found that recordings allowed the clinician to conduct the consultation without feeling the pressure of time or being scrutinised as well as minimising the chances of surreptitious recording and resultant legal action [8]. In some oncological cases, patients reported a significant memory barrier as being the result of a phenomenon known as chemotherapy-related cognitive impairment or ‘chemobrain,’ whereby there is an impairment in memory, concentration, attention, reasoning, visuospatial skills, and executive functioning [9–13]. This can last up to several years following chemotherapy [11, 14, 15]. In these oncological patients, personalised recordings of consultations were found to improve information retention and were used to help inform family and friends [16]. Generic recordings have been proposed as an option but Hack’s group found in their work in breast oncology that patients tended to prefer a personalised audio-recording of their own consultation instead [17]. In line with this, oncology researchers have now designed a consultation audio-recording mobile app which is currently being tested [18]. Although the majority of research in this area is in the field of cancer, studies have also been performed in orthopaedic [19] and neurosurgical [20] consultations where the results of qualitative interviews found recordings had a positive impact on patient understanding. Post-mastectomy breast reconstruction consultations differ from oncology, in that they relate to quality of life and its varying domains including psychosocial and sexual well-being, rather than issues of survival. These patients are in a very different frame of mind and can be at high risk of experiencing difficulties with memory after completing their chemotherapy treatment. For this reason, this study focused on patients receiving additional information specifically about breast reconstruction options. Furthermore, the impact of age on memory needs to be considered, as people later in life may have poorer information recall when compared to those who are younger [21, 22]. The aim of this study was therefore to use qualitative interviews to investigate the acceptability and value of audio-recordings as a memory aid in the delivery of information for pre-operative breast reconstruction patients.

## Methods

### Study design and participant groups

This was a prospective qualitative cohort study. All patients attended a pre-operative breast reconstruction consultation where they received detailed information relating to their surgery. Patients were allocated into two groups, recording or standard information package. Both groups were given written literature. The recording group received the written information but were additionally offered the opportunity to make an audio-recording of their consultation, on their own device. All patients were asked to participate in individual semi-structured interviews after their consultations. The researchers provided support for each of the participants in the recording group on how to record, save and access the audio-recordings on their personal devices. This process was estimated to take between 1 and 5 min.

Ethical approval for this study was obtained from the Southern Adelaide Clinical Human Research Ethics Committee (SAC HREC EC00188), OFR Number: 226.18.

### Participant recruitment

Participants were recruited over a 12-month period from a tertiary hospital, Flinders Medical Centre in South Australia. Participants were eligible if they had previously undergone a mastectomy or were scheduled for a reconstructive consultation in the context of a planned mastectomy for either breast cancer or high risk status and were considering breast reconstruction surgery. Participants were only excluded if their primary language was not English or if they did not have access to an iPhone or android device with recording capacity and data storage of at least 40 MB.

We aimed to recruit 40 participants, based on the number of participants thought to reach conceptual density with regard to emerging themes, whereby a sufficient depth of understanding is able to be reached to allow the researcher to theorise [3, 23–25]. This meant that the following criteria needed to be met: (1) a wide range of evidence was drawn from the data to illustrate the concepts, (2) the concepts were part of a rich network of themes in the data within which there are complex connections, (3) subtlety in the concepts is understood by the researcher and used constructively to articulate the richness in its meaning, (4) the concepts have resonance with existing literature in the area being investigated, and (5) the concepts, as part of a wider analytic story, stand up to testing for external validity [23]. The range criterion and the complexity criterion were satisfied using the computer aided qualitative analysis software NVivo which produced multiple instances of codes using line by line coding. The subtlety and ambiguity of concepts was facilitated in particular through memo writing, whereby the language used by participants in the research and the language used in the conceptual categories generated during the data analysis was analysed and examined. Criterion four was satisfied with a literature search which was undertaken prior to study design to explore which themes had already emerged and to test the sufficient conceptual depth of hypotheses. In order to satisfy criterion five, the researchers interpreted the findings in relation to the literature which has already been published and therefore expressed in terms which can be understood by those who have familiarity with this field of research.

### Interviews

Interviews were conducted at least a week prior to surgery, over the phone or face-to-face in a private room at Flinders Medical Centre, at a time that was convenient for the participant. Interviews were semi-structured, with both open and closed questions which allowed for an insight into participants’ thoughts and allowed them the freedom to explore ideas. Before commencing the interviews, participants were informed that if they would rather not answer any questions for any reason, then they could skip them, and could conclude the interview at any point. The interviews were audio-recorded with the participant’s permission. This allowed the interviewer to focus on the discussion and use the recording for transcription and detailed analysis at a later stage. The interviews were conducted by the same two researchers in order to limit interrater variability.

There were two sets of questions depending on the participant’s allocated group (see Additional file 1: Table S1). Participants were asked about their breast reconstruction consultation experience, the extent to which they thought recording their consultation was helpful or useful, and if they felt that this improved their understanding of the information received. Participants were also asked about their views on whether there should there be a role for recording consultations in the wider medical field and whether there were any other methods that could be employed to help patients feel like they were in a good place to make decisions about their surgery. Interview length was recorded but not time limited.

### Analysis

Interviews were transcribed verbatim by the research team from the recordings made and patient identifiers were removed. Following a framework analysis outline as described by Gale et al., coding of the transcripts was conducted by two members of the research team, independently [26]. This framework built on ideas which had already been established in the literature about some of the benefits and risks of recording consultations. These same two researchers met up after the first three transcripts and agreed on a set of codes to apply to the rest of the transcripts. These were then presented back to the research team to reach agreement in analysis and presentation of data. Data was coded using NVivo software (NVivo12 for Mac, QSR International).

## Results

Between 21/2/19 and 19/3/20, 32 women were recruited to the study, 17 were allocated into the recording group and 15 into the standard information package group. Two participants withdrew as they were no longer considering surgery and two could not be contacted and were considered lost to follow-up. The resulting 28 (16 recording and 12 standard information package) were interviewed and their transcripts analysed. There was only one situation where a participant lost access to their recording as it was not saved correctly during the consultation. Her responses were analysed according to the recording group, which she was allocated to.

The mean age of participants was 52 years (range 31–79 years). The majority (22/28) of the participants were married or in a de facto relationship. All of the participants were of Caucasian ethnicity. The age distribution as well as the timing and type of breast reconstruction is shown in Table 1.

**Table 1:** Study population demographics

Patients (n = 28)				
Age (years)	Total	Recording	No recording	
30–39	4	3	1	
40–49	10	5	5	
50–59	5	2	3	
60–69	5	4	1	
70–79	4	2	2	
Reconstructive procedure and timing	Total	Delayed	Immediate	Mixed
Latissimus dorsi ± expander	7	5	2	0
Free tram	3	1	1	1
Insertion of tissue expanders	7	1	4	2
Has not had surgery to date	11	n/a	n/a	n/a

Themes that emerged from the interview data are summarised in Additional file 2: Table S2 (see Additional file 2: Table S2) and included the following: positive experience, multiple revisions to refresh knowledge and understanding, usefulness in other specialist appointments, benefits to plastic surgeons, helps to inform family, home environment better for listening, impact on decision making and relationship with their surgeon. Each theme is further expanded with direct quotes from the participants, characterised by age and whether they had access to the recording.

### Theme 1: positive experience

All the participants, whether they had access to a recording or not, were in complete support of the idea of recording the explanatory part of consultation. The most frequently cited reason for this support was because of memory issues and ease of access to information.

### Theme 2: multiple revisions to refresh knowledge and understanding

It was well recognised by the participants that consultations can be overwhelming and many patients felt overloaded with information, even more so if they had limited health literacy or experience with doctors and surgeons. However, one participant noted that even if the patient was a health professional themselves and may understand more than others, it would still be beneficial for them to have a recording.

A key finding within this theme was that all participants recognised that they were likely to forget a proportion of the information that was conveyed in the consultation, so a recording could be used as a memory tool. It was interesting to note that despite the age of the participants, each was worried about their ability to retain and remember information, especially if they remarked that they had ‘chemo brain.’

Participants in the recording group reported listening to the recording between 1 and 3½ times. Older participants in this group tended to listen to their recording more times than participants who were younger (Table 2).

**Table 2:** The average amount of times participants listened to the audio recording of their consultation, by age

Patient age	Average number of times listened to the recording
	Lower–upper range (mean)
Ages: 30–39	1.67–1.67 (1.67)
Ages: 40–49	1.40–1.80 (1.60)
Ages: 50–59	2.50–2.50 (2.50)
Ages: 60–69	2.50–2.75 (2.63)
Ages: 70–79	2.50–3.50 (3.00)
All participants	2.00–2.31 (2.16)

### Theme 3: usefulness in other specialist appointments

Another common theme in interviews was that this sort of recording practice would be useful for other specialist appointments, particularly those which involved an explanation of detailed knowledge or difficult concepts. Most participants expressed that they would have appreciated an opportunity to record their oncological and breast surgery appointments, especially when they were first told their diagnosis. However, the consensus was that it was not necessary to record all doctors’ appointments, such as GP visits, unless there was a lot of information given or significant results were discussed.

### Theme 4: benefits to plastic surgeons

Interestingly there were only a few participants who considered the potential medico-legal implications for doctors. Other participants noted that they felt that it actually would increase their trust in their health care professional if they were willing to be recorded, so it could in fact be beneficial for the clinicians’ relationships with their patients. Similarly, some participants felt that plastic surgeons would also benefit from their patients feeling more comfortable as they would be more knowledgeable with information from their recording. It was also suggested that by recording consultations, plastic surgeons might be able to spend less time answering questions they’d already been asked, and could better use their time elsewhere.

### Theme 5: helps to inform family

Many of the participants explained that they preferred to listen to the recording at least once on their own but were happy for family members or close friends to listen to the recording if they wished. Family was very important to all of the participants, particularly those with close relationships with their husbands, parents and adult children (daughters in particular). Many participants thought that it was vital for their loved ones to listen to the recording too. This was especially the case if the family members missed the consultation or had a lot of questions which the participant couldn’t remember the answer to or found it difficult to explain.

### Theme 6: home environment better for listening

Another important aspect of the audio-recordings was the environment in which participants would feel most comfortable listening to them. All participants felt they could better absorb the information they needed at their own pace, by listening to the recording in their own home, where they felt comfortable and at ease.

### Theme 7: impact on decision making

There was a mixed response in terms of the impact of recordings on decision making about breast reconstruction procedures. Most participants already had an idea about the type of surgical outcomes that they wanted but conceded that their decision perhaps may have been better informed or reaffirmed.

### Theme 8: relationship with surgeon

Most patients thought their opinion of their plastic surgeon was not influenced by the option to record the consult. However, one participant, who had access to a recording, noted that they felt that they developed a closer relationship with their surgeon from hearing their voice multiple times. Another also remarked that if they had access to a recording, they felt that they would better understand the decisions and reasoning of their plastic surgeon.

## Discussion

The data collected in this study provided an insight into the value of recording pre-operative breast reconstruction consultations for breast cancer patients. There was a positive response to the innovation which has been previously found to be beneficial in a wide variety of consultation types [16, 19, 20, 27–29]. Our study found that participants were very interested in the idea of recording most types of consultations in which they were provided with complex information. One of the barriers to understanding information was health literacy. We found that the participants who had a background in health were more comfortable with receiving complex health information, but recognised that many others may not be. Many participants reported feeling overwhelmed and overloaded with information during their consultation. They experienced difficulties retaining and recalling information after the consultation and some attributed this to ‘chemobrain’ or chemotherapy-related cognitive impairment. Memory recall problems were reported by participants across the whole of the age spectrum. An audio-recording could be used as a memory aid whereby patients are able to go back over information and refresh their understanding. Indeed it has been previously established by Dommershuijsen and colleagues that one of the primary values of recording consultations is to increase patient recall [30].

Advice on treatment is commonly given in the stressful environment of a clinic, yet we have found that patients who had a recording of their consultation felt more comfortable reviewing this information in their own home environment and could better absorb the information they needed. This is consistent with Fleming and colleagues’ previous research on the home environment for people with memory problems with them finding that the practice promoted privacy and dignity [31]. It also follows Kessel’s article on the provision of a recording enabling patients to review their personalised information at home, with less anxiety and distress, which in turn allowed them to retain and absorb the information at their own pace [22].

Despite there being a consistently positive patient response to the idea of audio-recordings of clinical consultations, they have not yet become a mainstream part of the process. This may be because of several underlying concerns held by clinicians. These concerns relate to the risk of audio-recordings being used for purposes other than those for which they were made, such as for litigation in cases of dissatisfied or disgruntled patients. van Bruinessen and colleagues reported several concerns that were held by clinicians [32]. These included misuse of the recording via social media, uncertainty about the medico-legal status of the recordings, and worry that listening to a recording from a particular point in time may lead patients to adhere too rigidly to the wording and interpret things incorrectly later on in the time course of their treatment [32]. A recent article by Rimmer showed that during legal proceedings, audio-recordings were found to support the actions of clinicians rather than the patient’s claims [33]. Our study found that only a small number of participants brought up the idea of litigation of their own volition. Instead, we found that the recordings aided in building trust and confidence in the participant, as they felt that the surgeon had nothing to hide and was being open and honest. Nevertheless, clear medico-legal regulations may need to be put into place so that clinicians can be confident that literal statements said during a consultation could not be used against them in a legal dispute [32].

In addition, many participants agreed that having an audio-recording in other specialist settings, outside of pre-operative breast reconstruction consultations would be of benefit to them, particularly consultations where detailed knowledge or difficult concepts were discussed and more recall was required. These same sentiments were reported in a randomised cluster trial by Wolderslund and colleagues, who also found that audio-recordings positively influenced patient’s perception of having adequate information [34]. Several other studies have also found that an audio-recording was useful in specialist medical settings [19, 20].

One of the limitations of this study was that this was a single surgeon study at a single site, meaning that results may not be generalisable. Whilst the surgeon in this study was skilled and confident in their communication skills, this might not be the case for all health professionals. A recording of poor communication that does not address an individual patient’s information needs could be problematic.

Further limitations were that all the participants spoke English as their first language. This could have introduced a bias towards particular themes which were more important for English speaking participants from the particular socioeconomic areas which were located around the tertiary centre. Finally, all participants needed to have a working mobile phone device which was capable of recording a consultation and a researcher present to instruct them on how to use the recording application. Our study also relied on the availability of staff to explain to each participant how to use the recording feature on their own device, which may not be possible in other settings.

## Conclusion

This study has established that patients value access to a recording of the explanatory part of their breast reconstruction consultation and that their attitude towards audio-recordings as an aid in information delivery was positive. The audio-recording was utilised as a memory aid which could be used in the comfort and privacy of the patient’s own home. Data from interviews also supported the idea that the practice could be useful beyond the sphere of breast reconstruction, potentially being useful in any specialist appointment where extensive and complicated information is being given. Despite there still being some concern about the medico-legal implications of these recording, we found they can be a tool for building greater patient rapport and improving patient understanding of the pre-operative information received.

## Supplementary Information

**Additional file 1**. Table S1: Qualitative questionnaire

**Additional file 2**. Table S2: Recurring themes and supporting quotes

## Data Availability

The data that support the findings of this study are available from [Associate Professor Dr Nicola Dean] but restrictions apply to the availability of these data, which were used under license for the current study, and so are not publicly available. Data are however available from the authors upon reasonable request and with permission of [third party name—Associate Professor Dr Nicola Dean].
