# Supplementary material for: Improving preoperative breast reconstruction consultations: a qualitative study on the impact of personalised audio-recordings
Source: BMC Womens Health. 2021 Nov 6;21:389. doi: 10.1186/s12905-021-01534-8 (PMC8571820; doi:10.1186/s12905-021-01534-8)
Supplement: Supplementary file 1 — Additional file 1. Table S1: Qualitative questionnaire [file 12905_2021_1534_MOESM1_ESM.pdf]

TABLE 1

Qualitative questionnaire

Intervention Group:

1. What were your original thoughts about recording consultations? What are your thoughts now that you've had access to the recording? Did you have any hesitations about recording your consultation?
2. How do you think surgeons would feel about this idea?
3. Can you tell us a little bit about what the consultation was like?
4. Have you ever had a consultation that you wished you could record? Why/why not?
5. Can you please describe to us, if any, the expectations that you had from the recording? Did you find that the recording did or did not meet these expectations? Why do you think that is?
6. Can you please tell us if you found the recording helpful or useful in assisting you with your understanding of the surgical procedure? If so, in what why? If not, then why not?
7. How many times did you listen to the recording? Can you please describe the kind of environments that you were in and if anyone was with you? (If anyone was with you, what is the relationship between you and the other person, e.g., friend, partner, son, daughter? Was there a reason for why you wanted this person there, and did you find it was good/helpful etc?)
8. What impact do you think that your recording had on your decision making process, and on your relationship with your medical practitioner?
9. Following your consultation, did you have any further questions regarding the surgical procedure? Did you listen to the recording to help answer any questions? Why/why not? After listening back to the consultation, did you feel you knew everything you needed to know?
10. If you had unresolved questions, what do you think you'll do to get answers to these?
11. Would you recommend recorded consultations to other patients? Why/why not?
12. Do you think that recording consultations would be useful in other kinds of medical/surgical consultations for you? If so, then why and which types? If not, then why not?
13. Is there anything else you can think of that would help you be in a good position to know everything to make a good decision about your surgery?
14. Do you have any questions that you would like to ask us or any information that you would like to add?

Control Group:

1. What are your thoughts about recording consultations and would you have any hesitations about recording your consultation? Have you ever had a consultation with your doctor/ surgeon that you wished you could record? Why/why not?
2. How do you think surgeons would feel about this idea?
3. Can you tell us a little bit about what the consultation was like?
4. Following your consultation, did you have any further questions regarding the surgical procedure? Do you think that listening to a recording of your consultation would help answer any questions? Why/why not?
5. If you had unresolved questions, what do you think you'll do to get answers to these?
6. Have you ever had a consultation, surgical, general or any other kind, that you wished you could record? Why/why not?
7. What impact do you think being able to listen back to your recording might have?
8. How many times do you think you would listen to the recording? Can you please describe the kind of environments that you would be in and if anyone would be with you? (If anyone would be with you, what is the relationship between you and that person, eg, friend, partner, son, daughter?)
9. What impact do you think that your recording could have on your decision making process, and on your relationship with your medical practitioner?
10. Do you think that recording consultations would be useful in other kinds of medical/ surgical consultations for you? If so, then why and which types? If not, then why not?
11. Can you think of anything else that would aid in your understanding and decision making process for your surgical procedure?
12. Do you have any questions that you would like to ask us or any information that you would like to add?
