# Supplementary material for: Improving preoperative breast reconstruction consultations: a qualitative study on the impact of personalised audio-recordings
Source: BMC Womens Health. 2021 Nov 6;21:389. doi: 10.1186/s12905-021-01534-8 (PMC8571820; doi:10.1186/s12905-021-01534-8)
Supplement: Supplementary file 2 — Additional file 2. Table S2: Recurring themes and supporting quotes [file 12905_2021_1534_MOESM2_ESM.pdf]

Additional Table 2: Recurring themes and supporting quotes.

THEMES	SUPPORTING QUOTATIONS	SOURCE
**Positive experience**	*'I think it's a positive thing because it can be quite overwhelming for someone to take all that information in, especially depending on your educational level and your experience.'*	51 year-old, no recording)
	'I actually think that it's a brilliant idea, I really do… I think it's a really good innovation, if it ever came to being as general practice, I think that would be so useful to so many people…I think it's an awesome suggestion and that you are able to implement that.'	68 year-old, no recording
	'Yes I would, I think it would be very beneficial… This is a really good idea to do and I think that it's the way forward.'	55 year-old, recording
**Multiple revisions to refresh knowledge and understanding**	*'I think it's a really good idea because it just reinforces everything you're saying in the interview. You can just go over it and recap and think about what you've said and what was the information given to you, you have more options.'*	61 year-old, recording
	'I thought it was absolutely fantastic for the simple reason is that when you are given a whole heap of information and you're overwhelmed…it's really good to be able to go back to it whenever you need to and to keep updated with everything that's going on. It's improved my understanding, definitely. Because I've got it there if I need to… just to be able to go back to it and listen to it again.'	41 year-old, recording
	'I thought it was a good idea because me personally I forget a lot of things and especially in the moment because it's so much to take in, you forget a lot when you leave. 'Oh what did they say?' Because it's such a big thing, I find that it's overwhelming and I forget a lot. Then, with chemo as well, I have really bad 'chemo brain' so I forget a lot because of the chemo as well.'	31 year-old, recording
**Usefulness in other specialist appointments**	*'Especially in the realm of the cancer type things, you're under such stress and worry about what's happening to you, that you don't always absorb what's been told to you at the time because your mind is racing a thousand times a minute…I think most surgeries for anyone, it would be positive for anyone because it is an overwhelming thing to have to deal with sometimes.'*	51 year-old, no recording
	'I think they all should be recorded, especially when it comes to your own personal health and you just want the results and you want to be clear about them.'	61 year-old, recording
	'With my breast surgeon when I was first diagnosed… I just wish it was recorded because then I could have understood it all a little bit more – all the terminology and everything else. You get friends and family asking you questions about what they said but I don't know, I was in shock and I didn't have my husband with me.'	60 year-old, recording
	'It would have been nice to record my consultation with my breast surgeon…it would definitely be useful for chronic diseases and illnesses.'	50 year-old, no recording

**Benefits to plastic surgeons**	*'I think they'd probably be for it. They'd have less people ringing them I'd say to ask questions.'*<br>*'I think I would value my surgeon a lot more if they were prepared to be recorded and accountable.'*<br>*'I actually think that it's a brilliant idea, I really do. From both perspectives really – when a doctor sees probably 40 people a day, it would be wonderful for them to have a record of the interview to refresh their memory and any doubts about anything, but also I think it's very helpful to participants as well, so I think that it's an excellent idea.*	49 year-old, recording<br>44 year-old, no recording<br>68 year-old, no recording
**Helps to inform family**	*'My husband has been asking me a million questions and it would have been easier for me to play it for him.'*<br>*'Definitely my mum, yeah probably my dad and my husband probably... I'd be happy listening to it in front of them to explain some stuff to them if they needed to know.'*<br>*'I have with family and whilst my partner was with me. Whoever wants to listen to it, I'll definitely play it, so they've got more of an understanding of what I'm going through and what's happening. Because it is really hard to explain, it's a complete different language.'*	51 year-old, no recording<br>36 year-old, no recording<br>41 year-old, recording
**Home environment better for listening**	*'I'd be at home, because I could listen to it properly. I wouldn't be distracted by anything else and some of it I wouldn't necessarily want everyone else hearing so yep just do it at home.'*<br>*'At home where it's nice and peaceful, quiet. I think you take in the information better that way.'*	43 year-old, recording<br>55 year-old, recording
**Impact on decision making**	*'Would have made my decision making quicker and I think that it would have improved, would have strengthened my relationship.'*<br>*'I'd already decided about the procedure with my husband and knew it was going to be a big operation. I suppose it helped to clarify a bit more the stages of what's going on. I wouldn't say that listening back on it made me change my mind, because I'd already decided that it was the way forward.'*	44 year-old, no recording<br>55 year-old, recording
**Relationship with surgeon**	*'You just feel that you get to know someone a little bit more when you hear their voice several times on a recording. Makes me feel more comfortable next time I see them.'*<br>*'No I don't think it would change anything. I have great faith in my practitioners and specialists and it wouldn't change a thing.'*<br>*'I think we'd have a greater understanding of each other and how we both worked. How they worked as a doctor and my thoughts on what I thought about it as well. Maybe the communication side of it would be more efficient.'*	74 year-old, recording<br>51 year-old, no recording<br>47 year-old, no recording
